# Thoracic duct obstruction leading to chylous mesenteric cyst in an female obese adolescent: a case report

**DOI:** 10.1097/MS9.0000000000003267

**Published:** 2025-04-10

**Authors:** Xiao-Zhou Li, Zi-Hao Deng, Xin Deng, Qing Zhang, Jing Wang, Tian-Xiong Li, De-Xiao Du, Bu-He A-Min, Hao Zhao

**Affiliations:** a Surgery Centre of Diabetes Mellitus, Capital Medical University Affiliated Beijing Shijitan Hospital, Beijing, China; b Capital Medical University, Beijing, China; c Department of Lymphatic Surgery, Capital Medical University Affiliated Beijing Shijitan Hospital, Beijing, China; d Department of Anesthesiology, Capital Medical University Affiliated Beijing Shijitan Hospital, Beijing, China

**Keywords:** adolescent, chylous mesenteric cyst, low-fat diet, obesity, thoracic duct obstruction

## Abstract

**Introduction and importance:**

Mesenteric cysts are rare intra-abdominal lesions, with a prevalence of approximately 1 in 100 000 to 1 in 250 000 adults. Clinical presentation can range from asymptomatic to severe. Chylous mesenteric cysts caused by thoracic duct obstruction are particularly rare and have not been previously reported in obese adolescents.

**Case presentation:**

We report the case of a 19-year-old female patient with a rare asymptomatic chylous mesenteric cyst caused by thoracic duct obstruction, which was incidentally discovered during pre-operative evaluation for bariatric surgery. The patient underwent thoracic duct decompression and a personalized low-fat nutrition plan was implemented to mitigate postoperative chyle reflux and address concurrent obesity.

**Clinical discussion:**

Mesenteric cysts can have variable clinical presentations and their evaluation relies on a range of imaging modalities. Our case highlights the importance of lymphatic system evaluation in the diagnosis and management of mesenteric cysts, particularly chylous cysts. Surgery targeting lymphatic system abnormalities offers a targeted and less invasive treatment strategy than the traditional surgical resection.

**Conclusion:**

This case report emphasizes the importance of imaging evaluation of the lymphatic system for the diagnosis and management of mesenteric cysts. A treatment strategy that combines surgery targeting lymphatic system abnormalities with a personalized nutritional plan can provide a novel and less invasive alternative to traditional surgical resection of chylous mesenteric cysts, addressing the underlying etiology while minimizing surgical trauma and potential complications.

## Introduction

Mesenteric cysts are rare intra-abdominal lesions with a prevalence of approximately 1 in 100 000 to 1 in 250 000 adult cases and 1 in 20 000 pediatric cases^[1,2]^. These cysts were first described by the Italian anatomist Benevieni in 1507 during the autopsy^[2,3]^. Cysts can present as single or multiple fluid-filled cysts containing serous, hemorrhagic, purulent, or chylous fluid. Most of these cysts are benign, with a malignancy rate of 3%^[4]^.

**HIGHLIGHTS**

- Mesenteric cysts can be asymptomatic and can be discovered incidentally.
- Lymphangiography plays an important role in the diagnosis and evaluation of mesenteric cysts, particularly in identifying lymphatic system abnormalities.
- In cases where cysts are caused by lymphatic system abnormalities, targeted surgical intervention may be an effective alternative to total cyst resection, addressing the underlying etiology while minimizing surgical trauma.
- A multidisciplinary approach combining lymphatic surgery and nutritional planning can offer a targeted and less-invasive treatment strategy for mesenteric cysts caused by lymphatic system abnormalities.

While most mesenteric cysts are asymptomatic, some patients may present with symptoms, such as abdominal pain, nausea, vomiting, and intestinal obstruction. The severity of these symptoms depends on the size, location, and associated complications of the cysts. Surgical resection is generally considered as the primary treatment for mesenteric cysts to prevent recurrence and malignant transformation. In select cases, alternative approaches, such as aspiration, cyst fenestration, or marsupialization, may be considered, although these methods carry a higher risk of recurrence.

The current study reports a rare case of an asymptomatic chylous mesenteric cyst caused by thoracic duct obstruction in an obese patient. To the best of our knowledge, to date, there have been no reports of similar cases. This case report is in line with SCARE Criteria^[5]^.

## Presentation of case

A 19-year-old Asian female university student with no personal income and financial dependence on family support presented for metabolic bariatric surgery, with a history of significant weight gain over 4 years, increasing from 80 kg to a peak of 110 kg, now stabilized at 105 kg with a BMI of 39.6 kg/m^2^, waist circumstance of 127 cm. Weight gain was associated with increased food intake and the frequent consumption of takeout meals. She reported no notable symptoms or discomforts. The patient had been diagnosed with hypertension and hyperuricemia 1 year prior. Her family history was significant for malignancies: her grandfather had died of pancreatic cancer, her aunt had been diagnosed with a malignant pericardial tumor, and her uncle had been diagnosed with leukemia.

**Figure 1.**
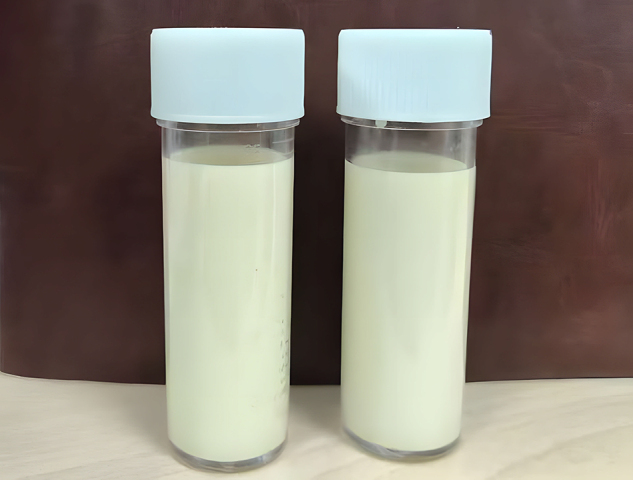
Pre-operative CT scans of mesenteric cysts. Pre-operative CT scan demonstrating multiple cystic lesions in the right abdomen and pelvis, with the largest lesion measuring 22.34 cm × 13.89 cm, characteristic of mesenteric cysts in the present case.

On physical examination, abdominal distension was observed, but no palpable masses, tenderness, or muscle rigidity was detected. Laboratory investigations revealed elevated triglycerides (TG, 1.78 mmol/L; reference <1.7 mmol/L) and decreased high-density lipoprotein cholesterol (HDL-C, 0.99 mmol/L; reference 1.10–1.74 mmol/L). Tests for autoimmune markers, including p-ANCA, c-ANCA, MPO, PR3, ANA, and dsDNA, were negative. Other routine blood test results revealed no significant abnormalities. The aspirated fluid exhibited a chylous appearance (Fig. 1), confirming its classification as chylous fluid, as summarized in Table 1. From the perspective of psychological and quality-of-life assessments, her SAS (Self-Rating Anxiety Scale) score was 53 and SDS (Self-Rating Depression Scale) score was 57, both falling within the mild symptom range. Additionally, her IWQOL-lite (Impact of Weight on Quality of Life-Lite) score of 112 indicated moderate-to-severe impairment in daily functioning and psychosocial well-being due to obesity-related concerns.

**Table 1:** Cystic fluid analysis results

Parameter	Result
Gross appearance	Chylous
Sudan III stain	Positive
Specific gravity	>1.050
Nucleated cell count	0.961 × 10^9^/L (99% mononuclear cells)
Total protein	82.4 g/L
Lactate dehydrogenase (LD)	220 U/L
Adenosine deaminase (ADA)	8.5 U/L
Total cholesterol (TC)	5.47 mmol/L
Triglycerides	2.85 mmol/L

**Figure 2.**
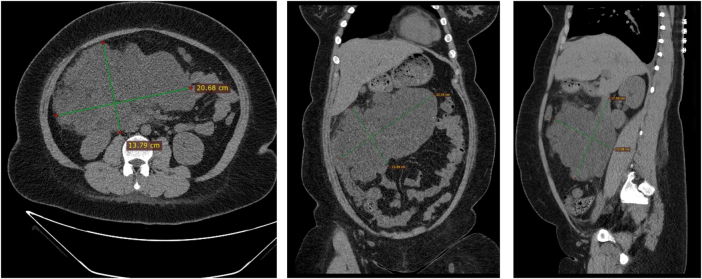
MR lymphangiography of chylous mesenteric cysts. MR lymphangiography demonstrated irregular cystic long T2 signals in the right lower abdomen with relatively clear boundaries, indicating the presence of chylous mesenteric cysts and obstruction of chylous flow at the terminal thoracic duct.

**Figure 3.**
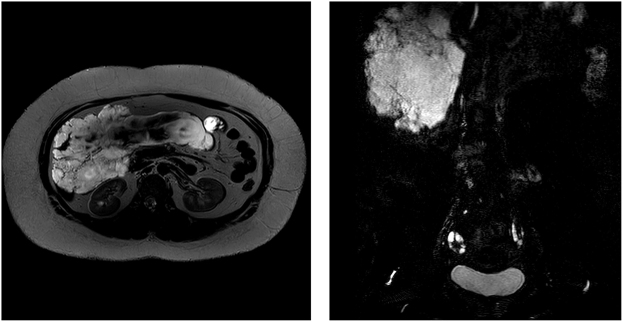
Aspirated fluid shows chylous appearance. A chylous appearance was noted in the aspirated fluid, which tested positive for Sudan III.

Contrast-enhanced abdominal computed tomography (CT) was performed after a routine plain CT scan and revealed multiple cystic lesions in the right abdomen and pelvis with a CT attenuation value of approximately 13 HU. The lesions exhibited a lobulated appearance, extending along the mesenteric-pancreatic space and partially reaching the right retroperitoneum. The borders were poorly defined with close proximity to the surrounding intestinal loops and encasement of the ascending colon. The largest lesion measured 22.34 cm × 13.89 cm (Fig. 2). Magnetic resonance (MR) lymphangiography revealed irregular cystic long T2 signals in the right lower abdomen with relatively clear boundaries (Fig. 3). Lymphangiography also indicated obstruction of chylous flow in the terminal thoracic duct (Supplemental Video, http://links.lww.com/MS9/A794).

On the basis of the patient’s medical history, imaging findings, and cystic fluid analysis, an abdominal chylous cyst was diagnosed. The patient was subsequently referred to the lymphatic surgery department for decompression of the thoracic duct. During surgery, the thoracic duct was identified at its confluence with the left jugular, subclavian, and bronchomediastinal trunk. Dense fibrous tissue, fatty tissue, and the vascular sheath of the left internal jugular vein compress the thoracic duct and ampulla, causing luminal narrowing and chylous reflux. The compressive structures were carefully dissected and excised to restore chylous flow into the venous angle. After release, the thoracic duct and the associated lymphatic trunk showed improved chylous drainage without reflux. The thoracic duct and lymphatic trunk diameters were measured: thoracic duct, 3.8 mm; bronchomediastinal trunk, 1.0 mm; jugular trunk, 1.8 mm; and subclavian trunk, 0.5 mm. Sodium hyaluronate was used to prevent adhesions. Histopathological examination of the excised tissue revealed fibrous and fatty tissues with collagen deposition.

**Figure 4.**
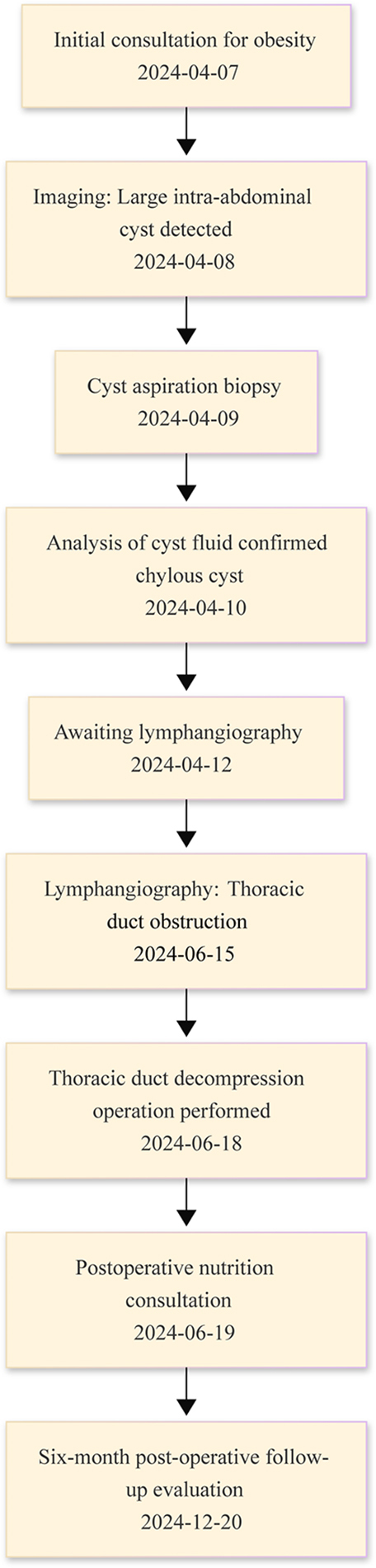
The timeline of patient’s diagnosis and therapy.

Nutritional consultation was conducted to mitigate postoperative chyle reflux and to address concurrent obesity. The nutrition department provided a personalized long-term low-fat diet plan with a daily caloric intake of 1200 kcal. Supplementation with compound vitamin B tablets, administered at a dose of two tablets three times daily, was initiated to ensure adequate micronutrient intake. Additional supplements, including multivitamin, Vitamin D3, probiotic and prebiotic compound preparations, and high-purity fish oil or DHA algae oil, were prescribed at standardized doses. Following a three-day postoperative hospital stay, during which the patient remained stable and complication-free, discharge was facilitated. Subsequent multidisciplinary follow-up care was provided by our center with regular assessments to monitor the patient’s progress and adjust the treatment plan as needed. Fig. 4 presents the patient’s diagnostic and therapeutic timeline.

## Discussion

Mesenteric cysts are a rare entity characterized by variable clinical presentation, ranging from asymptomatic to severe manifestations, including rupture and abdominal infection. As summarized in Table 2, previous studies have documented the clinico-temporal profiles and management approaches of mesenteric cysts. Symptom severity is influenced by the cyst size, location, and associated complications^[6,7]^. Common presenting symptoms included abdominal pain reported in 82% of cases, nausea and vomiting in 45%, constipation in 27%, and diarrhea in 6%, with a palpable abdominal mass detected in up to 61% of patients^[8]^. However, a significant proportion (approximately 40%) of mesenteric cysts are discovered incidentally during routine examinations under unrelated conditions^[9,10]^. This is illustrated by the present case, in which the mesenteric cyst was incidentally discovered during pre-operative evaluation for bariatric surgery.

**Table 2:** Clinico-temporal profiles of mesenteric cyst cases with intervention details

Author	Year	Gender	Age	Case	Chief complaint	Treatment
Ferreira et al.	2018	Male	20	Large mesenteric cyst	Abdominal distension	Laparotomy was performed to remove the cyst completely
Ward et al.	2011	Female	25	A ruptured infected mesenteric cyst diagnosed on laparoscopy for suspected appendicitis	24 hours worsening generalized abdominal pain with fever malaise and anorexia	Diagnostic laparoscopy was performed. The left iliac fossa stab incision was lengthened to 40 mm, creating a mini-laparotomy incision to enable resection of the effected length of small bowel and a hand-sewn end-to-end bowel anastomosis was performed.
Pithawa et al.	2014	Male	7	Mesenteric cyst: A rare intra-abdominal tumor	Progressive enlargement of the left abdominal mass with dull left abdominal pain for 5 months	Exploratory laparotomy was performed. Complete enucleation of cyst carried out leaving the large intestine intact
Singh et al.	2023	Female	4	Mesenteric cyst: A rare entity	Abdominal pain lasting 4 days, accompanied by black stools for 2 days, poor oral feeding	The patient underwent laparotomy for the excision of the mesenteric cyst with adherent small bowel resection and anastomosis
Mason et al.	2001	Case 1: Female. Case 2: Female	Case 1: 32 Case 2: 66	Laparoscopic excision of mesenteric cysts	Case 1: A painless mass in the upper left abdomen case2: progressive epigastric pain radiating to right subcostal region for 6 mounth and gastroesophageal reflux	Case 1: Laparoscopic exploration and excision of the mass were performed. case2: Laparoscopic exploration, excision of the mass, and laparoscopic Nissen fundoplication were performed.
Gagliardi et al.	2022	Female	55	Mesenteric cyst with GI symptoms: A fluid approach to treatment-case report and literature review	Left abdominal constipation discomfort for three months	Laparoscopic surgery to remove the cyst without requiring any bowel resection
Bhattacharjee et al.	2022	Female	70	A rare case of a mesenteric cyst	Abdominal pain for 1 month with constipation for 15 days	Significant adhesions were removed by blunt dissection, partial drainage of the mesenteric cyst content was performed
Falidas et al.	2011	Male	45	Traumatic mesenteric cyst after blunt abdominal trauma	Upper abdominal pain was accompanied by abdominal distention, and the abdominal mass gradually increased for 5 months	Complete resection and removal of the mass
Cudia et al.	2020	Male	74	Lymphatic mesenteric cyst, a rare cause of surgical abdominal pain	Diffuse abdominal pain for 2 month	They performed a complete open surgical excision of the lesion, which was located in the mesentery of the ileal loop.

The evaluation of mesenteric cysts relies on a range of imaging modalities, including ultrasound, CT, and MRI, which provide distinct insights into cyst characteristics. On ultrasonography, mesenteric cysts typically present as well-defined anechoic masses with possible septations or debris^[11]^. CT scans are useful for assessing cyst size and extent, whereas MRI is particularly valuable for evaluating complex features such as multiloculated structures or the presence of fat within the cyst contents, and can aid in differentiating between cyst types based on signal characteristics^[12]^.

The etiology of mesenteric cysts is multifarious and is potentially linked to aberrant lymphatic system development^[13]^, particularly structural or functional anomalies such as complex lymphatic anomalies (CLAs) that disrupt chylous drainage via malformations (e.g., thoracic duct obstruction) or valvular incompetence, leading to elevated lymphatic pressure and cystic chyle accumulation^[14,15]^. For instance, benign proliferation of lymphatic tissue that fails to interface with the lymphatic system, or factors such as trauma, infection, and tumors that precipitate lymphatic obstruction, lymph node degeneration, ectopic lymphoid tissue proliferation, or mesenteric leaf fusion failure^[16,17]^. However, the precise causative mechanism remains unclear. Considering the predominance of chylous cysts, a more focused approach to lymphatic system evaluation is warranted in clinical practice to identify treatable targets (e.g., thoracic duct stenosis) and stratify therapies. In this context, our use of MR lymphangiography in the present patient revealed obstruction of the chylous flow at the terminal thoracic duct, providing insight into the underlying etiology and directly guiding the selection of targeted interventions such as thoracic duct embolization or stent-graft decompression, which resolve pressure gradients and reduce cyst recurrence^[18,19]^, underscoring the diagnostic value of lymphangiography in mesenteric cyst evaluation.

Given the patient’s significant body mass and the massive intra-abdominal cyst occupying the operative field, laparoscopic resection was deemed unfeasible due to restricted visualization, while open surgery posed risks of substantial trauma and postoperative fat liquefaction[20]. After multidisciplinary evaluation, we prioritized a minimally invasive strategy. Rather than performing total resection of the cysts in the present patients, we opted for thoracic duct decompression, which was performed in collaboration with the lymphatic surgery department, and complemented by a personalized nutrition plan from the nutrition department. Although the recurrence rate is relatively low as 0–15% as reported[9], it does not address the underlying etiology of cysts and carries significant surgical trauma. By contrast, our approach offers a more targeted and less invasive treatment strategy that minimizes potential complications.

This case report has limitations in that it is a single-case study, and the long-term efficacy and potential complications of thoracic duct decompression for chylous mesenteric cysts require further investigation in large-scale studies. Additionally, the patient’s concurrent obesity and metabolic improvements following nutritional management may have influenced the treatment outcome, making it challenging to isolate the effects of thoracic duct decompression alone. Furthermore, the cyst remains unresected, carrying risks of recurrence and malignant transformation, particularly given the patient’s family history of malignancy, necessitating long-term surveillance.

## Conclusion

In conclusion, this case report highlights the importance of imaging evaluation of the lymphatic system for the diagnosis and management of mesenteric cysts. Our treatment strategy, which combines thoracic duct decompression with a personalized nutritional plan, offers a novel and less invasive alternative to traditional surgical resection that addresses the underlying etiology while minimizing surgical trauma and potential complications.

### Patient perspective

**Figure 5.**
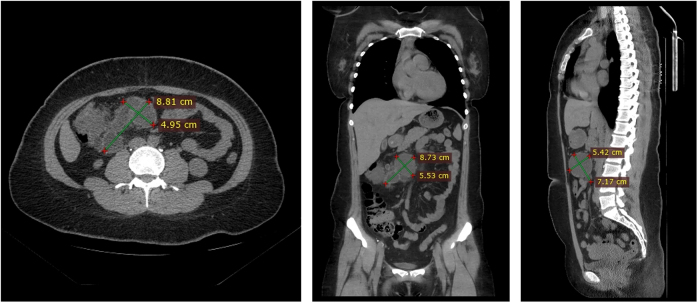
Post-operative CT scans of mesenteric cysts. Post-operative CT scan demonstrates a substantial reduction in cyst size, measuring 8.73 cm × 5.53 cm, following thoracic duct decompression and nutritional management, indicating successful treatment of the chylous mesenteric cyst.

At the six-month postoperative follow-up, the patient has followed the recommended dietary plan quite well and demonstrated excellent overall health and remarkable weight loss of 30 kg (BMI reduced to 28.6 kg/m^2^, waist circumference reduced to 102 cm), accompanied by significant metabolic improvements. Notably, her psychological assessments showed marked improvement, with SAS and SDS scores declining to 48 and 50, respectively, and her IWQOL-lite score improving to 74, reflecting reduced anxiety/depression symptoms and enhanced quality of life. Laboratory tests confirmed normalization of key metabolic parameters. Imaging studies revealed a substantial reduction in cyst size, with the lesion measuring 8.73 cm × 5.53 cm (Fig. 5), showing no complications or abnormalities.

## Data Availability

Given the nature of the data sharing is not applicable to this article.
